# Predicted Risks of Cardiovascular Disease Following Chemotherapy and Radiotherapy in the UK NCRI RAPID Trial of Positron Emission Tomography–Directed Therapy for Early-Stage Hodgkin Lymphoma

**DOI:** 10.1200/JCO.21.00408

**Published:** 2021-08-13

**Authors:** David J. Cutter, Johanna Ramroth, Patricia Diez, Andy Buckle, Georgios Ntentas, Bilyana Popova, Laura Clifton-Hadley, Peter J. Hoskin, Sarah C. Darby, John Radford, Tim Illidge

**Affiliations:** ^1^ Nuffield Department of Population Health, University of Oxford, Oxford, United Kingdom; ^2^ Oxford Cancer and Haematology Centre, Oxford University Hospitals NHS Foundation Trust, Churchill Hospital, Oxford, United Kingdom; ^3^ National Radiotherapy Trials Quality Assurance Group, Mount Vernon Cancer Centre, Northwood, United Kingdom; ^4^ Department of Medical Physics, Guy's and St Thomas' NHS Foundation Trust, London, United Kingdom; ^5^ Cancer Research UK, UCL Cancer Trials Centre, London, United Kingdom; ^6^ Manchester Academic Health Science Centre, Manchester Cancer Research Centre, University of Manchester, The Christie NHS Foundation Trust, Manchester, United Kingdom

## Abstract

**PURPOSE:**

The contemporary management of early-stage Hodgkin lymphoma (ES-HL) involves balancing the risk of late adverse effects of radiotherapy against the increased risk of relapse if radiotherapy is omitted. This study provides information on the risk of radiation-related cardiovascular disease to help personalize the delivery of radiotherapy in ES-HL.

**METHODS:**

We predicted 30-year absolute cardiovascular risk from chemotherapy and involved field radiotherapy in patients who were positron emission tomography (PET)–negative following three cycles of doxorubicin, bleomycin, vinblastine, and dacarbazine chemotherapy within a UK randomized trial of PET-directed therapy for ES-HL. Cardiac and carotid radiation doses and chemotherapy exposure were combined with established dose-response relationships and population-based mortality and incidence rates.

**RESULTS:**

Average mean heart dose was 4.0 Gy (range 0.1-24.0 Gy) and average bilateral common carotid artery dose was 21.5 Gy (range 0.6-38.1 Gy), based on individualized cardiovascular dosimetry for 144 PET-negative patients receiving involved field radiotherapy. The average predicted 30-year radiation-related absolute excess overall cardiovascular mortality was 0.56% (range 0.01%-6.79%; < 0.5% in 67% of patients and > 1% in 15%), whereas average predicted 30-year excess incidence was 6.24% (range 0.31%-31.09%; < 5% in 58% of patients and > 10% in 24%). For cardiac disease, the average predicted 30-year radiation-related absolute excess mortality was 0.42% (0.79% with mediastinal involvement and 0.05% without) and for stroke, it was 0.14%.

**CONCLUSION:**

Predicted excess cardiovascular risk is small for most patients, so radiotherapy may provide net benefit. However, for a minority of patients receiving high doses of radiation to cardiovascular structures, it may be preferable to consider advanced radiotherapy techniques to reduce doses or to omit radiotherapy and accept the increased relapse risk. Individual assessment of cardiovascular and other risks before treatment would allow personalized decision making about radiotherapy in ES-HL.

## INTRODUCTION

Over recent decades, the standard management of early-stage Hodgkin lymphoma (ES-HL) has been combined modality treatment including chemotherapy and radiotherapy. For favorable ES-HL, this is currently two cycles of doxorubicin, bleomycin, vinblastine, and dacarbazine (ABVD) chemotherapy and 20 Gy involved field radiotherapy (IFRT), giving excellent 5-year survival (> 90% relapse-free and > 95% overall).^1^ Attention is now focused on reducing late toxicity. In the past, extended-field radiotherapy and higher radiation doses provided good disease control but incurred substantial risks of second cancers and cardiovascular disease (CVD).^2,3^ More recently, randomized controlled trials (RCTs) have combined clinical risk factors and positron emission tomography (PET)—a radiologic biomarker of response—to identify patients for whom initial treatment can be less intensive, hopefully reducing long-term toxicity without compromising cure.^1,4,5,6^

**CONTEXT**

**Key Objective**
To predict 30-year absolute excess risks of radiation-related cardiovascular disease for positron emission tomography–negative patients given radiotherapy in the UK RAPID trial of early-stage Hodgkin lymphoma using novel methodology that combines individual radiation dosimetry and epidemiologic data.
**Knowledge Generated**
Mean heart dose was < 1 Gy for more than half the patients and < 5 Gy for more than two thirds, whereas mean bilateral common carotid dose was 21 Gy. If radiotherapy were given selectively to the 50% of patients with the lowest predicted risks, then their average predicted 30-year absolute excess risks of radiation-related cardiovascular disease would be 0.11% for mortality and 1.79% for incidence.
**Relevance**
For the majority of positron emission tomography–negative patients in the RAPID trial, the predicted cardiovascular risks are small. These risks should be even lower with the most modern radiotherapy techniques. The decision to give radiotherapy for early-stage Hodgkin lymphoma should be patient-specific, based on individualized risk predictions of dose to critical structures and of all the late effects of these exposures.

The UK National Cancer Research Institute Lymphoma Study Group RAPID trial was an RCT in ES-HL designed to test the omission of radiotherapy following a complete metabolic response on fluorodeoxyglucose-PET scans after three cycles of ABVD. Patients achieving Deauville score^7^ 1-2 were randomly assigned either to no further treatment (NFT) or to IFRT. Considering all randomly assigned patients, 3-year progression-free survival (PFS) did not differ between the two groups (NFT 90.8% *v* IFRT 94.6%, 3.8% absolute reduction, *P* = .16). However, considering only the patients who received their allocated treatment, the benefit of radiotherapy was significant (NFT 90.8% *v* 97.1%, 6.3% absolute reduction, *P* = .02).^5^ Further evidence for radiotherapy benefit was reported for the EORTC/LYSA/FIL H10 and GHSG H16 Trials.^4,6^ While PFS is appropriate for assessing the efficacy of Hodgkin lymphoma (HL) treatment over 3-5 years, an overall assessment of the effect of radiotherapy needs to take late toxicity into account, balancing a proven improvement in initial disease control versus radiotherapy toxicities that may occur beyond 5-10 years and compromise long-term health and survival.^2^

Among HL survivors, cardiac death was, historically, the leading cause of mortality associated with radiotherapy exposure of a single organ.^2,8^ While cardiac doses are lower with IFRT than extended-field radiotherapy,^9,10^ no detailed description of doses received by individual cardiac substructures with IFRT has been published, nor have any predictions been made of the resulting cardiovascular risks for an RCT cohort.

The aim of this study is to quantify the cardiovascular radiation doses received by ES-HL patients given IFRT within a recent RCT and to use them to predict the absolute risk of radiation-related CVD. Greater understanding of these risks would improve a personalized approach to the contemporary use of radiotherapy.

## METHODS

### Patients and Treatment

Six hundred two patients were enrolled into the trial during 2003-2010 (median age, 34 years; range, 16-75 years). Five hundred seventy-one received three cycles of ABVD followed by fluorodeoxyglucose-PET. Among PET-negative individuals (Deauville score 1-2, n = 426), 209 were randomly assigned to IFRT and 183 received it. Among PET-positive individuals (Deauville score 3-5, n = 145), 129 received IFRT (Data Supplement, online only).

IFRT comprised treating the extent of disease detected by computed tomography (CT) before chemotherapy. There was no intention to treat uninvolved contiguous nodal areas, or entire nodal regions. The recommended field-edge margins were 5 cm up-and-down involved nodal chains with 1.5-2.0 cm lateral margins around the postchemotherapy volume of disease within the mediastinum.^11^ The dose specified was 30 Gy in 1.8-2.0 Gy fractions, treated in the supine position, by opposed anterior and posterior 5-8 MV beams, both delivered daily.

### Cardiovascular Radiation Dosimetry

Radiotherapy departments supplied details of the IFRT administered to the National Radiotherapy Trials Quality Assurance team. Where CT planning was used (72%), the original data sets were requested. If the CT did not cover the entire heart, regression was used to estimate the volume missing to calculate heart dose and to estimate doses to cardiac substructures. Where x-ray simulation was used (28%), copies of films were requested and substitute CTs were used to estimate dosimetry. Further details are in the Data Supplement. Treatment data collection and subsequent analysis were approved by the appropriate Research Ethics Committee.

### Prediction of Cardiovascular Risks

Predictions of 30-year cardiovascular mortality were based on individual radiation doses to the whole heart, left ventricle, heart valves, and common carotid arteries, and the administered anthracycline dose. They were derived using the estimated percentage increases in mortality rate per unit dose obtained from long-term studies of cardiac disease^12-14^ and stroke^15^ following HL treatment, combined with 5-year age- and sex-specific death rates from CVD in the general UK population. Deaths from all causes other than CVD in the general population were competing risks. The 30-year risk of incident CVD was estimated in a similar fashion, using age- and sex-specific first CVD incidence rates from a representative UK cohort^16^ (details are in the Data Supplement).

## RESULTS

### Patients Included in the Analysis

Of the 183 PET-negative patients who received IFRT, data sufficient to complete dosimetry were available for 144 (78.7%, Data Supplement). Of the 129 PET-positive patients who received IFRT, data sufficient to complete dosimetry were available for 103 (79.8%). The baseline characteristics of patients for whom dosimetry was and was not completed were similar (Data Supplement).

### Doses Received by the Heart, Cardiac Substructures, and Carotid Arteries

**FIG 1.**
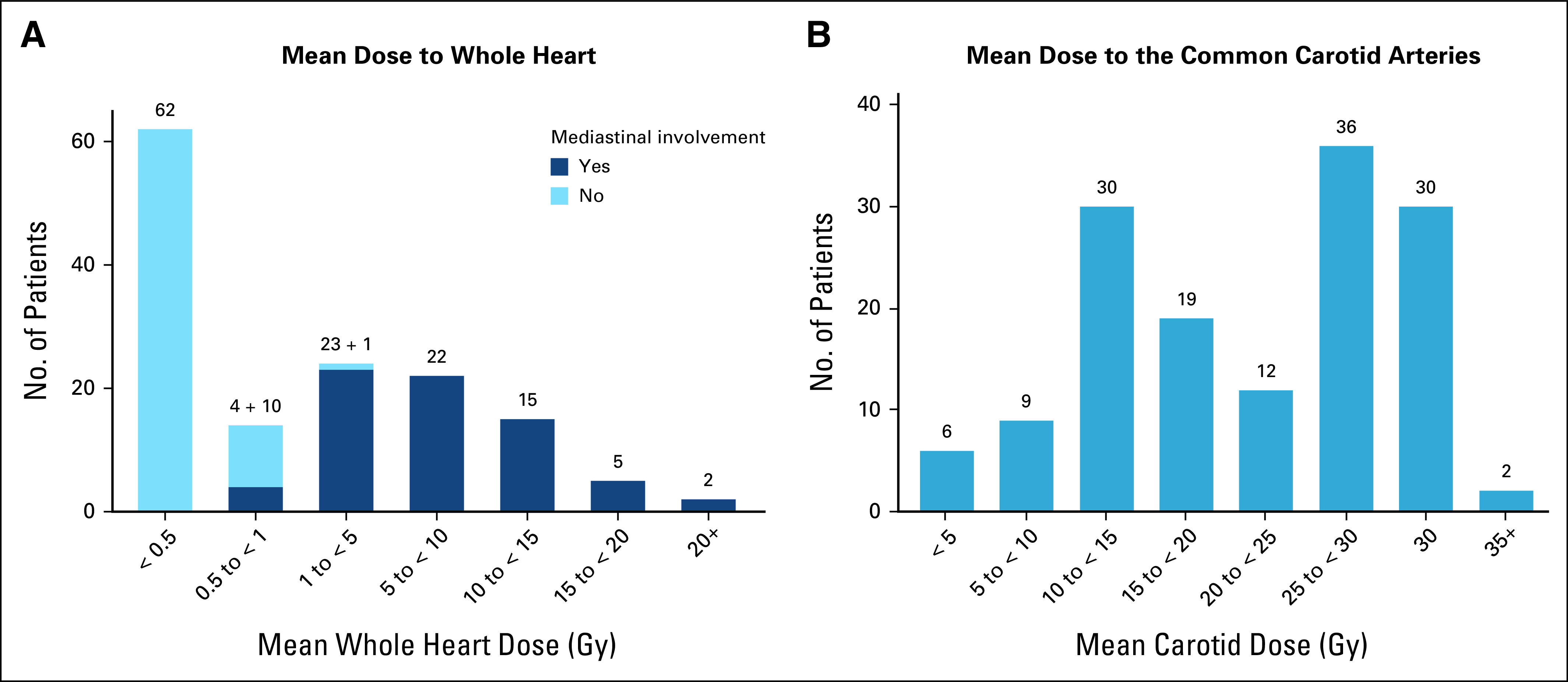
Distribution of mean doses in Gy to cardiovascular structures for PET-negative patients receiving radiotherapy and for whom dosimetry was completed (n = 144). (A) Whole heart, showing those with mediastinal involvement (dark blue) and those without (light blue). (B) Common carotid arteries. PET, positron emission tomography.

For PET-negative patients, the average MHD was 4.0 Gy: 0.3 Gy for those without and 7.8 Gy for those with mediastinal involvement (Table 1). For almost all patients without mediastinal involvement, the MHD was < 1 Gy (72/73, 98.6%), whereas for those with mediastinal involvement, the MHD ranged widely (0.8-24.0 Gy, Table 1 and Fig 1A). Considering all PET-negative patients, MHD was < 1 Gy for more than half (76/144, 52.8%) and < 5 Gy for more than two thirds (100/144, 69.4%). The most superior cardiac substructures, such as the pulmonary valve and sinoatrial node, received the highest mean radiation doses (Table 1). The mean radiation dose to the common carotid arteries averaged over 20 Gy but varied widely with peaks for patients receiving unilateral and bilateral neck irradiation (Fig 1B). Dose distributions for PET-positive patients receiving IFRT were similar to those with PET-negative disease (Data Supplement).

**TABLE 1.**
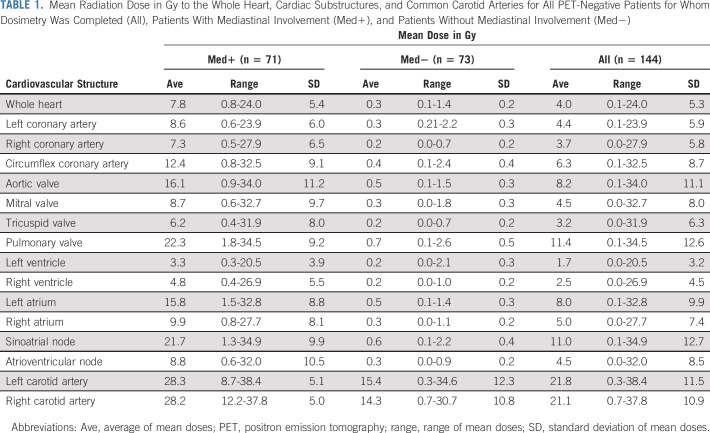
Mean Radiation Dose in Gy to the Whole Heart, Cardiac Substructures, and Common Carotid Arteries for All PET-Negative Patients for Whom Dosimetry Was Completed (All), Patients With Mediastinal Involvement (Med+), and Patients Without Mediastinal Involvement (Med−)

### Predicted 30-Year Risks of Cardiovascular Mortality

#### Mortality from heart disease or stroke.

**FIG 2.**
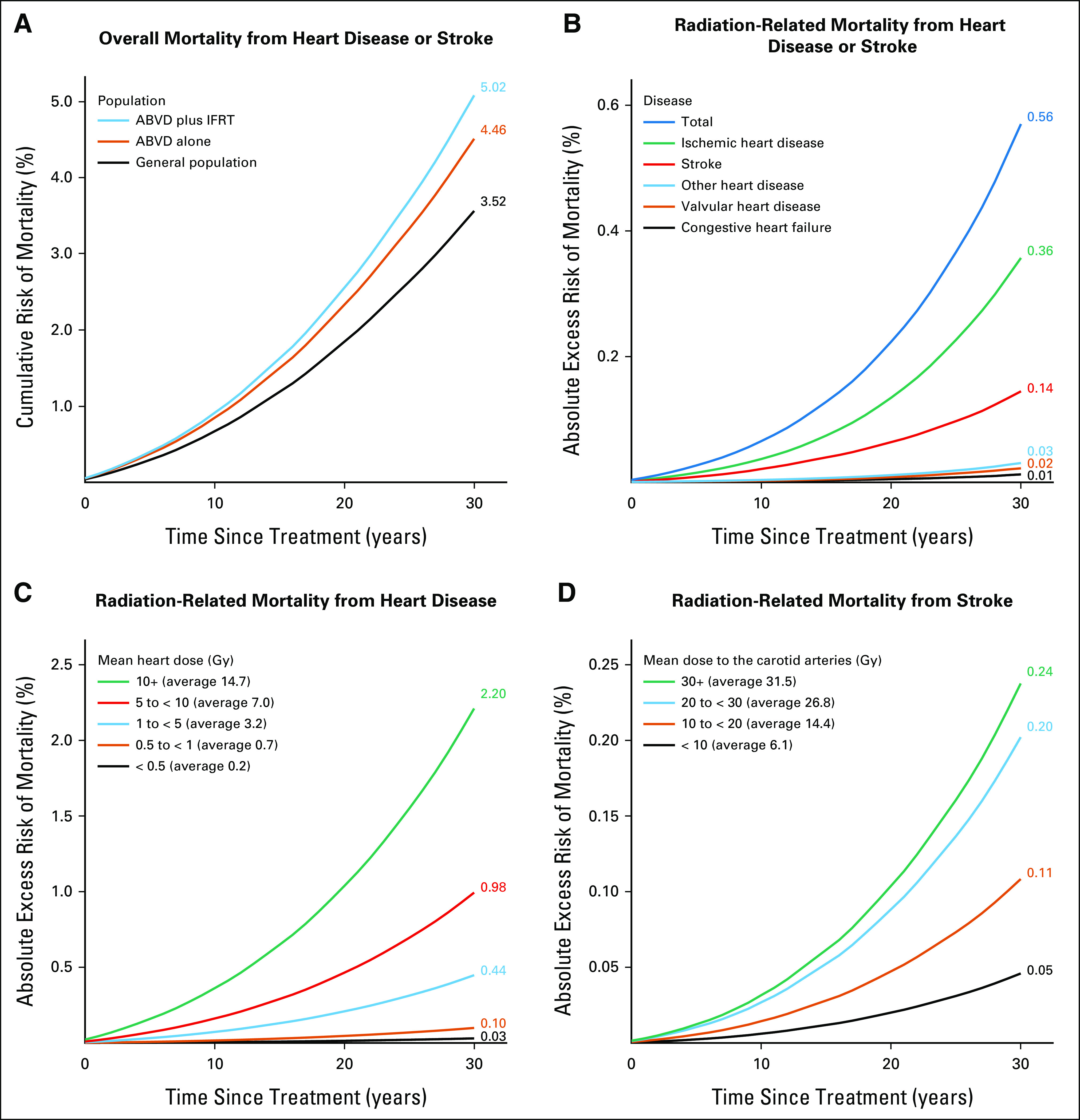
Predicted cardiovascular mortality for PET-negative patients receiving radiotherapy for whom radiation dosimetry was completed (n = 144). (A) Predicted cumulative risks of mortality from heart disease or stroke for the general population, for patients receiving ABVD chemotherapy alone, and for patients receiving ABVD plus IFRT. Cumulative risks allow for the competing risk of noncardiovascular causes of death. (B) Predicted absolute excess risk of mortality from heart disease or stroke for patients receiving ABVD plus IFRT compared with patients receiving ABVD alone, by disease category. (C) Predicted absolute excess risk of mortality from heart disease for patients receiving ABVD plus IFRT compared with patients receiving ABVD alone, by categories of mean whole heart dose in Gy. To achieve comparability across categories, predictions for each category assume all 144 patients received the average radiation dose in that category. (D) Predicted absolute excess risk of mortality from stroke for patients receiving ABVD plus IFRT compared with patients receiving ABVD alone, by categories of mean bilateral common carotid artery dose in Gy. To achieve comparability across categories, predictions for each category assume all 144 patients received the average radiation dose in that category. ABVD, doxorubicin, bleomycin, vinblastine, and dacarbazine; IFRT, involved field radiotherapy; PET, positron emission tomography.

**FIG 3.**
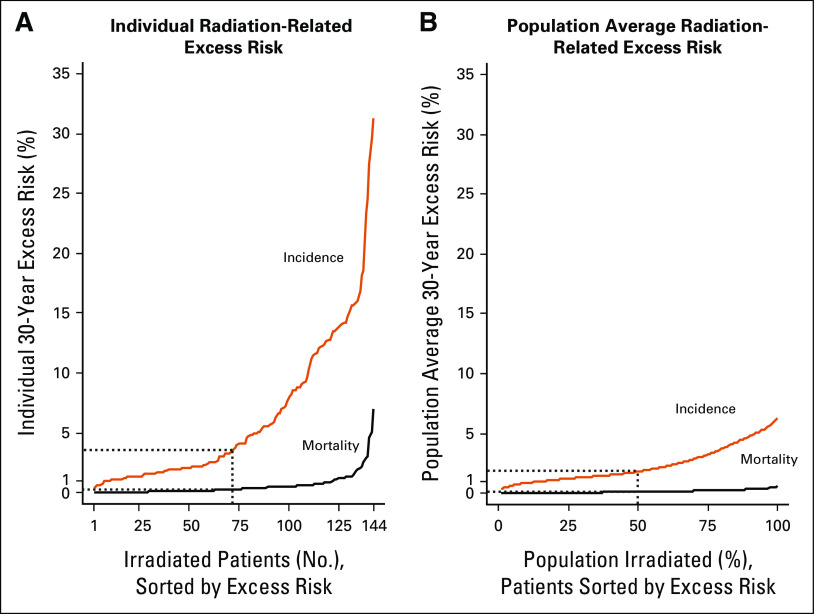
Predicted 30-year absolute excess risk of radiation-related heart disease or stroke for PET-negative patients who received radiotherapy and for whom dosimetry was completed (n = 144). Mortality is indicated by orange lines and incidence by black lines. (A) Individual absolute excess risks. Dotted line corresponds to the median excess risk (ie, average of patients ranked 72 and 73 of 144), which was 0.26% for mortality and 3.61% for incidence. (B) Population average absolute excess risks. Dotted line corresponds to population average for irradiated patients if 50% of PET-negative patients with the lowest predicted risks were irradiated. This group of irradiated patients has 0.11% average excess risk for mortality and 1.79% for incidence. PET, positron emission tomography.

The average predicted 30-year cardiovascular mortality risk for PET-negative patients who received IFRT after ABVD was 5.02% (range over individuals 0.30%-19.37%), and comprised 3.52% expected risk from general population rates plus 0.94% absolute excess risk because of anthracycline chemotherapy and a further 0.56% because of IFRT (Fig 2A). The absolute excess risk because of IFRT was dominated by ischemic heart disease (0.36%) and stroke (0.14%; Fig 2B). Considering the radiation-related risk to individual patients, the predicted 30-year absolute excess was < 0.5% in 67% of patients, whereas the median risk (ie, the average of patients ranked 72 and 73 out of 144) was 0.26%. The range across individuals was 0.01%-6.79% (Fig 3A) and the risk was > 1% in 15% of patients. If IFRT were given selectively to the 50% of PET-negative patients with the lowest predicted radiation-related risks, then the average predicted 30-year absolute excess radiation-related cardiovascular risk for these patients would be 0.11% (Fig 3B).

#### Mortality from heart disease.

When the PET-negative patients were subdivided into five categories of MHD, the average predicted 30-year absolute excess risk of radiation-related mortality from heart disease ranged from 0.03% for those receiving < 0.5 Gy MHD to 2.20% for those receiving 10+ Gy (Fig 2C). For individuals, the radiation-related risk ranged from 0.002% to 6.55%. The average was 0.42%; 0.79% for those with mediastinal involvement and 0.05% without. The main determinant of MHD, and hence of cardiac risk, was the inferior border of the radiotherapy field (Data Supplement). Average MHD was higher for females than for males (5.4 *v* 2.7 Gy) because of a higher proportion with mediastinal involvement (59% *v* 41%) and, on average, a lower inferior border to the mediastinal radiation field (median level seventh thoracic vertebra in females *v* sixth in males). Consequently, the predicted *proportional* increase in mortality from heart disease was on average higher for females. However, as men have higher cardiac mortality rates in the general population, the estimated 30-year *absolute* excess mortality risk from treatment-related heart disease (chemotherapy and radiotherapy combined) was actually lower for females (1.2% for females and 1.5% for males, Data Supplement).

#### Mortality from stroke.

When the PET-negative patients were grouped into four categories of mean bilateral carotid artery dose, the predicted 30-year average absolute excess radiation-related risk of mortality from stroke varied from 0.05% in those receiving < 10 Gy to 0.24% in those receiving 30+ Gy (Fig 2D). For individual patients, the radiation-related risk ranged from 0.008% to 1.12%, with an average of 0.14%.

### Predicted 30-Year Risks of Incident CVD

#### Incidence of heart disease or stroke.

**FIG 4.**
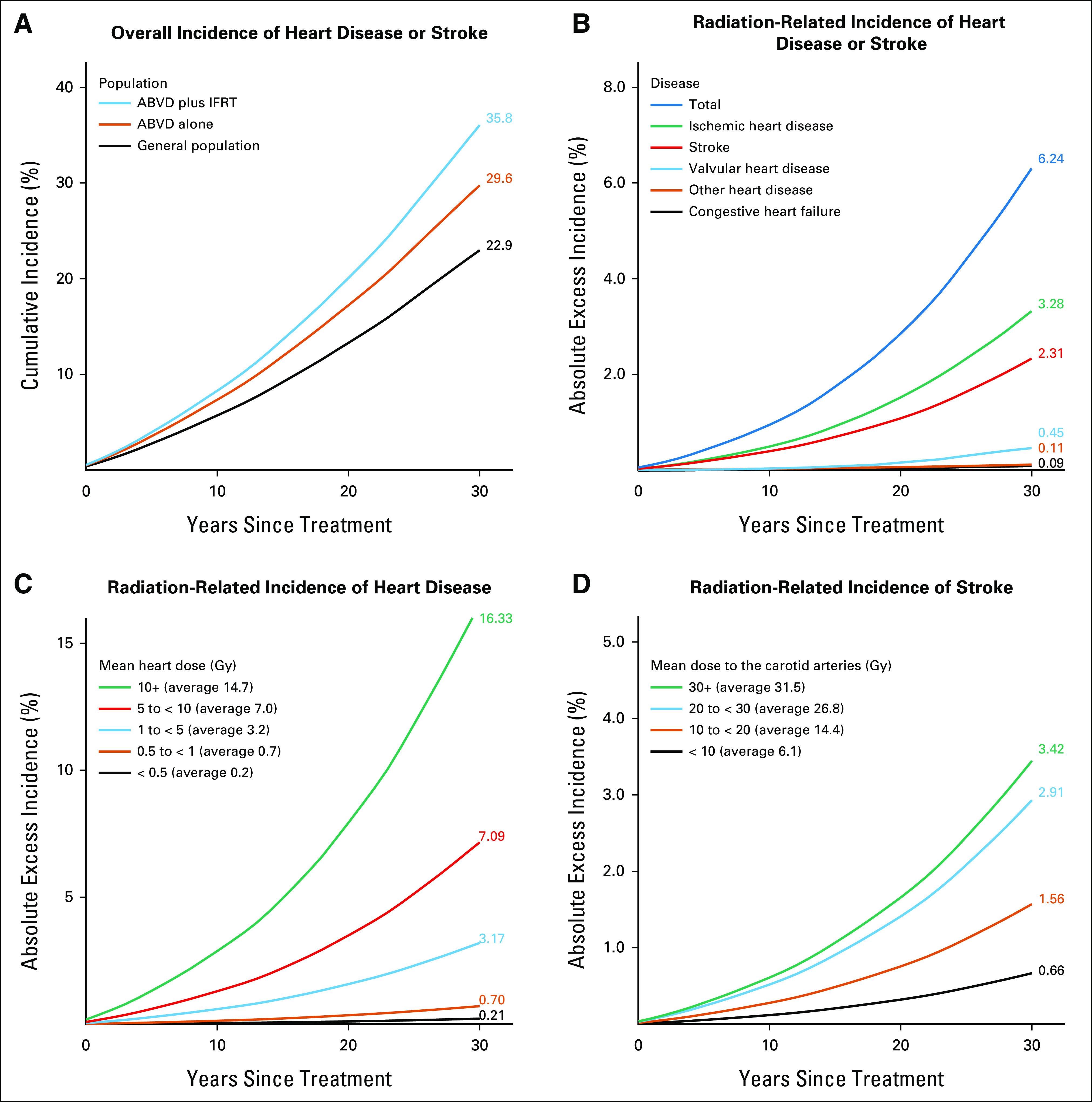
Predicted cardiovascular disease incidence for PET-negative patients receiving radiotherapy and for whom radiation dosimetry was completed (n = 144). (A) Predicted cumulative risks of incident heart disease or stroke for the general population, for patients receiving ABVD chemotherapy alone, and for patients receiving ABVD plus IFRT. Cumulative risks allow for the competing risk of noncardiovascular causes of death. (B) Predicted absolute excess risk of incident heart disease or stroke for patients receiving ABVD plus IFRT compared with patients receiving ABVD alone, by disease category. (C) Predicted absolute excess risk of incident heart disease for patients receiving ABVD plus IFRT compared with patients receiving ABVD alone, by categories of mean whole heart dose in Gy. To achieve comparability across categories, predictions for each category assume all 144 patients received the average radiation dose in that category. (D) Predicted absolute excess risk of incident stroke for patients receiving ABVD plus IFRT compared with patients receiving ABVD alone, by categories of mean bilateral common carotid artery dose in Gy. To achieve comparability across categories, predictions for each category assume all 144 patients received the average radiation dose in that category. ABVD, doxorubicin, bleomycin, vinblastine, and dacarbazine; IFRT, involved field radiotherapy; PET, positron emission tomography.

The average predicted 30-year risk of developing CVD for the PET-negative patients receiving IFRT after ABVD was 35.8% (range over individuals 7.7%-86.8%). This comprised 22.9% expected risk from general population rates plus 6.7% absolute excess risk because of anthracycline chemotherapy and a further 6.2% because of IFRT (Fig 4A). The absolute excess risk because of IFRT was dominated by ischemic heart disease (3.28%) and stroke (2.31%; Fig 4B). Considering the radiation-related risk to individual patients, the predicted 30-year absolute excess risk was < 5% in 58% of patients, whereas the median individual risk was 3.61%. The range across individuals was 0.31%-31.09% (Fig 3A) and the risk was > 10% in 24% of patients. If IFRT were given selectively to the 50% of PET-negative patients with the lowest predicted radiation-related cardiovascular risks, then the average predicted 30-year excess absolute radiation-related incidence risk for these patients would be 1.79% (Fig 3B).

#### Incidence of heart disease.

When individuals were grouped into five categories of MHD, the average predicted 30-year absolute excess risk of developing radiation-related heart disease ranged from 0.21% for those receiving < 0.5 Gy MHD to 16.33% for those receiving 10+ Gy (Fig 4C). For individuals, the radiation-related risk ranged from 0.03% to 27.88%. The average was 3.93%; 7.66% for those with mediastinal involvement and 0.31% without.

#### Incidence of stroke.

When individuals were grouped into four categories of mean bilateral carotid artery dose, the predicted 30-year absolute excess risk of incident stroke ranged from 0.66% in those receiving < 10 Gy to 3.42% in those receiving ≥ 30 Gy (Fig 4D). For individuals, the radiation-related risk ranged from 0.09% to 5.35%, with an average of 2.31%.

## DISCUSSION

This study reports the cardiovascular radiation doses received by ES-HL patients treated with IFRT within the RAPID trial and uses them to predict 30-year radiation-related cardiovascular risks for patients who were PET-negative after initial chemotherapy. Because of the varied distribution of disease, the doses received varied widely between individuals and so, therefore, did the predicted radiation-related risks. For 67% of patients, the predicted radiation-related 30-year absolute excess cardiovascular mortality risk was < 0.5% and for 58%, the incidence risk was < 5%. Although these risks are low, they are clinically relevant when considered in the context of an expected 5-year relapse-free survival of > 95% for ES-HL. At the other end of the scale, for 15% of patients, the mortality risk was > 1% and for 24%, the incidence risk was > 10%. For all patients, individualized late toxicity risks should be balanced against the 6%-12% absolute benefit in PFS from consolidation radiotherapy observed in three large RCTs.^4,5,6^

Our study gives a representative picture of cardiovascular risk from IFRT for all patients in the RAPID trial who were PET-negative after initial chemotherapy. Previous dosimetry studies have concentrated largely on patients with more extensive mediastinal involvement and reported techniques to reduce cardiac exposure.^9,17-20^ In this study, radiotherapy did not include the mediastinum for more than half the patients and we confirm previous findings that the level of mediastinal involvement is a critical determinant of cardiac dose, largely independent of the radiation techniques used.^17,21,22^ With our methods, the minority of patients receiving the highest radiation doses to the heart and carotid arteries have predicted risks of CVD similar to those seen for historical forms of radiotherapy (Table 2) and consistent with results observed from large cohort studies with prolonged follow-up.^15,23^ In sharp contrast are the 35% who received only unilateral neck irradiation in the RAPID trial for whom the doses to cardiovascular structures, and consequently the predicted risks, are much lower.

**TABLE 2.**
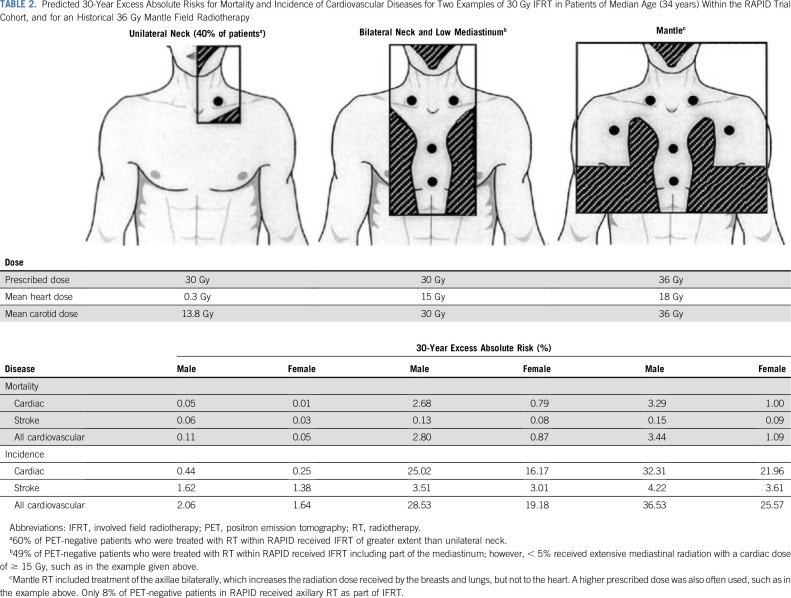
Predicted 30-Year Excess Absolute Risks for Mortality and Incidence of Cardiovascular Diseases for Two Examples of 30 Gy IFRT in Patients of Median Age (34 years) Within the RAPID Trial Cohort, and for an Historical 36 Gy Mantle Field Radiotherapy

Anthracycline-based chemotherapy approximately doubles the risk of cardiac disease from mediastinal irradiation, even in the presence of lower cardiac radiation doses,^24-26^ and it increases cardiac mortality risk even in the absence of radiotherapy.^27^ Anthracycline exposure was therefore included in the models used to predict treatment-related cardiac disease. It is important to recognize that the cardiac risk from chemotherapy in many patients is equal to, or greater than, the risk from radiotherapy. Indeed, we estimate that in the RAPID cohort, the cardiovascular risk from anthracycline exposure exceeded that from radiotherapy in 65% of individuals.

A strength of this study is the number of individuals (n = 247, 144 PET-negative) for whom dosimetry was completed, which is considerably greater than in the largest previous study reporting cardiac doses from IFRT (n = 41).^9^ Dosimetry was completed on a high proportion of patients receiving radiotherapy within the trial (79%), and comparison of baseline characteristics suggests that this sample is representative of the entire cohort. The study includes patients from 42 radiotherapy centers across the United Kingdom (87.5% of departments within the trial). It is therefore a representative sample of UK practice at the time.

There are, however, some limitations to this study. First, not all dosimetry was based on individual anatomy, as substitute CT data sets and data interpolation were used to calculate doses for a minority of patients (28%). This is unlikely to affect the average cardiovascular doses and predicted risks substantially, as demonstrated in a recent study.^28^ Second, the method used to predict radiation-related CVD has not been prospectively validated, as 20 additional years of follow-up would be required to assess the accuracy of the predictions. Our method is, however, based on the best available epidemiologic evidence regarding the magnitude of the long-term risks of radiation and anthracycline chemotherapy in HL survivors, together with current mortality and incidence rates in the general population. Third, we cannot provide separate risks for patients with pre-existing cardiovascular risk factors such as smoking and diabetes. In the future, adjustments to the model could use individual data on cardiovascular risk factors, but such information was not available for this study. Fourth, we did not attempt to model the possible impact of HL relapse in a small proportion of patients or the cardiotoxicity of subsequent treatments, so it is likely that we are underestimating the net benefit of initial radiotherapy. Finally, although we used the dose metrics from the best epidemiologic evidence currently available^12-15^ as the basis for our methods, we recognize the ongoing uncertainty over the radiation dose metric that may best predict the risks of radiation-related cardiac disease and stroke.^29,30^

The challenge of a personalized approach in ES-HL is to balance the risk of late adverse effects from radiotherapy against the omission of radiotherapy, which in turn increases risk of relapse and necessitates further therapy, perhaps including autologous stem-cell transplantation. If, rather than giving radiotherapy to all patients (or to none) regardless of their individual risk of late effects, radiotherapy were given just to patients predicted to be at lower risk of radiation-related CVD, then a substantial proportion of the benefit in terms of recurrence reduction would remain while fewer radiation-related cardiovascular complications would occur. For example, if the 50% of PET-negative patients at lowest risk of cardiovascular complications received radiotherapy, around 50% of the recurrences that would occur by withholding radiotherapy would be prevented, but only one fifth of the excess cardiovascular mortality from irradiating the whole cohort would be incurred (0.11%, Figs 3B, of the total 0.56%, Fig 2A). No individual patient's 30-year excess absolute cardiovascular mortality risk from IFRT would exceed 0.26% (Fig 3A) and the therapeutic ratio of the treatment would be improved. Current efforts to identify which individuals have greatest reduction in relapse risk from upfront radiotherapy (eg, using maximal tumor dimension at diagnosis^31^) may also help to identify patients who would benefit most from combined modality treatment.

It should be noted that developments in radiotherapy techniques since the RAPID trial, including smaller target volumes,^32^ the use of deep-inspiration breath-hold techniques,^33^ optimized intensity-modulated radiotherapy,^34^ and proton beam therapy,^18^ have reduced irradiated volumes and radiation doses to normal tissues, especially when combined with a 20 Gy prescribed dose for favorable ES-HL.^35^ Radiation-related CVD is not the only late side effect that may be reduced by the omission of radiotherapy. Risks of second cancers and other radiation-related late toxicities such as xerostomia and hypothyroidism, which would also be reduced by a chemotherapy-only approach, are also likely to be lower with contemporary radiotherapy, but a comprehensive assessment of late toxicities goes beyond the scope of this study. Consideration of all the important toxicities of alternative treatment strategies including the toxicity of treating possible HL relapse would likely suggest that an optimal approach will involve irradiating those ES-HL patients who are at lower risk of RT-related toxicity, rather than a strategy advising radiotherapy for all or none, based only on PET response.

In conclusion, IFRT for ES-HL as given in RAPID is likely to produce a small increase in the long-term risk of CVD. However, the magnitude of the risk varies widely and, for a majority of patients, the benefit of reduced HL relapse substantially outweighs the risk of CVD. With more modern radiotherapy techniques, the cardiovascular radiation doses achieved^35^ would result in even lower predicted risks than those seen for the RAPID cohort. As the sites of disease and degree of mediastinal involvement are known at diagnosis, the radiation doses to cardiovascular organs, and hence the risk of radiation-related CVD, can be estimated when the initial treatment strategy is decided. Such an approach could identify high-risk patients who, because of their predicted radiation-related cardiovascular risk, should be considered for treatment with chemotherapy alone as well as for novel radiation techniques, such as deep-inspiration breath-hold techniques and proton beam therapy, that can minimize cardiac exposure.^18,33^ As knowledge increases, a personalized assessment including all relevant risks would be helpful in determining the optimal strategy for individual patients.

## Data Availability

All data requests relating to the RAPID trial should be submitted for consideration to the trial sponsor via the Chief Investigator (john.radford@manchester.ac.uk). All data requests relating to the dosimetric and risk prediction analysis should be submitted to the corresponding author (david.cutter@ndph.ox.ac.uk). All information necessary to reproduce the calculation of cardiovascular risks is provided or referenced in the Data Supplement.

## Notes

**Johanna Ramroth**
**Employment:** Gilead Sciences (I) **Stock and Other Ownership Interests:** Gilead Sciences
